# Thermal ablation for ata intermediate-risk papillary thyroid carcinoma: efficacy and safety outcomes in a retrospective cohort

**DOI:** 10.3389/fendo.2025.1736498

**Published:** 2025-12-30

**Authors:** Tian-hao Cong, Ying Wei, Zhen-long Zhao, Shi-liang Cao, Na Yu, Jie Wu, Xin-yi Zhou, Han-xiao Zhao, Li-li Peng, Yan Li, Ming-an Yu

**Affiliations:** 1 Interventional Medicine, China-Japan Friendship Hospital, Beijing, China; 2 Beijing University of Chinese Medicine, Beijing, China; 3 China-Japan Friendship Institute of Clinical Medical Sciences, Beijing, China

**Keywords:** thermal ablation, intermediate-risk, papillary thyroid carcinoma, hydrodissection, ultrasound

## Abstract

**Purpose:**

To evaluate the efficacy and safety of thermal ablation (TA) for ATA intermediate-risk papillary thyroid carcinoma (PTC).

**Methods and materials:**

This retrospective study analyzed ATA intermediate-risk PTC (either ultrasound extrathyroidal extension or cervical lymph node < 3cm metastasis) patients treated with TA at China-Japan Friendship Hospital between April 2018 and December 2023. Outcomes included technical success, recurrence-free survival (RFS), and complications. Multi-Cox regression identified prognostic factors.

**Results:**

A total of 113 people were included. TA demonstrated 100% technical success and complete ablation rates. 18 patients (15.9%) had developed tumor recurrence. The 1-, 3-, and 5-year RFS rates were 95.6%, 83.7%, and 79.8%, respectively. Metastatic lymph node diameter independently predicted progression (HR:3.20, *p*<0.05). Complications occurred in 21.2% of cases, with 1.8% permanent vocal cord paralysis.

**Conclusions:**

TA shows promising efficacy and safety for selected ATA intermediate-risk PTC patients, with lymph node size as a key prognostic factor.

## Highlights

- Question: This study addresses the lack of evidence on the efficacy and safety of thermal ablation (TA) as an alternative to surgery for ATA intermediate-risk papillary thyroid carcinoma (PTC).
- Findings: Thermal ablation achieved 100% technical success, 79.8% 5-year recurrence-free survival, and a 21.2% complication rate (1.8% permanent vocal cord paralysis) in intermediate-risk PTC. Lymph node diameter as a key predictor of recurrence.
- Clinical Relevant Statement: Thermal ablation offers a minimally invasive, effective alternative to surgery for intermediate-risk PTC, preserving thyroid function while achieving durable cancer control (79.8% 5-year RFS) with lower complication risks (1.8% permanent vocal damage) and avoiding lifelong hormone replacement.

## 1 Introduction

Papillary thyroid carcinoma (PTC), the most prevalent subtype of differentiated thyroid cancer (DTC) (1), generally demonstrates favorable clinical outcomes due to its indolent tumor biology (2, 3). However, 8.2%-14.7% of patients experience disease recurrence or mortality associated with lymph node metastasis or extrathyroidal extension (4–6). Therefore, it is crucial to focus further on this patient population.

With the advancement of interventional thyroidology, thermal ablation (TA) techniques, including radiofrequency ablation (RFA) and microwave ablation (MWA), have been increasingly adopted for the treatment of thyroid cancer (7). Historically, TA was primarily utilized for recurrent thyroid carcinoma and solitary papillary thyroid microcarcinomas (PTMCs) (8–11). However, growing evidence suggests an expanding role of TA, with recent studies exploring its efficacy in larger tumors, multifocal lesions, and even select cases with extrathyroidal extension (12). These findings not only underscore the therapeutic potential of TA in thyroid cancer management but also reinforce its indispensable position in the current treatment paradigm.

The ATA Risk Stratification System, the most widely used postoperative risk prediction tool for thyroid cancer, is designed to assess the recurrence risk following surgical resection in thyroid cancer patients (13, 14). Notably, patients classified as intermediate-risk under this system face a substantial recurrence rate of 10%–30% (13, 14), which remains clinically suboptimal. Moreover, these patients often endure significant treatment-related burdens, including lifelong hormone replacement therapy, surgical scarring, and associated costs. However, the efficacy of TA for ATA intermediate-risk patients remains poorly defined, and whether TA can serve as a viable alternative for this population is yet to be established. To address this knowledge gap, we conducted a retrospective study of PTC patients who underwent TA at China-Japan Friendship Hospital between 2018 and 2023. The objectives were to evaluate the recurrence-free survival (RFS) and complication rates following TA in ATA intermediate-risk PTC patients.

## 2 Materials and methods

This study was conducted in accordance with the World Medical Association Declaration of Helsinki and was approved by the Institutional Review Board (IRB) of China-Japan Friendship Hospital. Written informed consent was obtained from all participants prior to treatment. Given the retrospective nature of this study, the IRB granted a waiver of additional informed consent for data collection and analysis.

### 2.1 Patient

We retrospectively enrolled patients with PTC who underwent TA as first-line treatment at China-Japan Friendship Hospital between April 2018 and December 2023.

The inclusion criteria were:

(1)Cytologically confirmed PTC via fine-needle aspiration (FNA);
(2) Selection of TA as primary treatment;
(3) Presence of either “ultrasound” extrathyroidal extension (uETE) or cervical lymph node metastasis (LNM) at initial diagnosis.

The exclusion criteria comprised:

(1) Death due to other malignant tumors during follow-up;
(2) Incomplete clinical data;
(3) Any metastatic lymph node measuring ≥3 cm or PTC node > 4 cm in maximum diameter at initial treatment;
(4) Macroscopic invasion.

**Figure 1:**
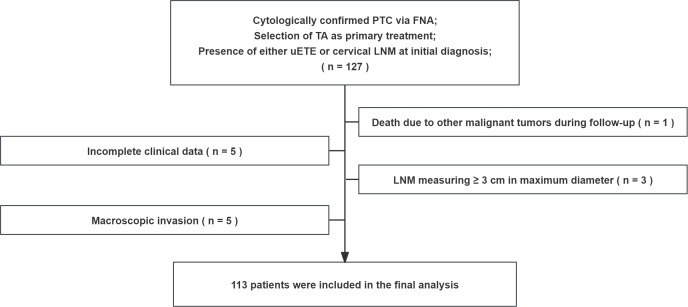
Study flowchart. FNA, fine-needle aspiration; uETE, ultrasound extrathyroidal extension; LNM, lymph node metastasis; TA, thermal ablation PTC, Papillary thyroid carcinoma.

After applying these criteria, 113 patients were included in the final analysis (**Figure 1**). All patients included in the study declined surgical intervention due to either medical contraindications (e.g., poor general condition precluding general anesthesia) or personal preference (such as the desire to preserve thyroid function or refusal to accept surgical scarring).

### 2.2 Preoperative and postoperative evaluation

All patients underwent comprehensive ultrasound (US) evaluation before and after ablation (Nanjing ECO Microwave System or Surgnova Radiofrequency Ablation System) using high-resolution US scanners GE LOGIQ E9 (GE Healthcare, USA) or Aplio 500 (Toshiba, Japan) equipped with linear transducers. The largest tumor or metastatic lymph node was identified as the index lesion. When extrathyroidal extension was present, the tumor with invasion was designated as the index lesion. Tumor characteristics were described based on the index lesion. All patients underwent comprehensive evaluation during and within 24 hours after the ablation procedure, including US, contrast-enhanced ultrasound (CEUS), and physical examination, to assess the ablation outcomes and detect potential complications. The procedure was immediately terminated if intolerable adverse reactions or complications occurred during treatment, and ablation was performed again after symptom resolution. Follow-up was conducted every 3 months in the first year, every 6 months from years 2 to 4, and annually thereafter. The follow-up contents include ultrasound examinations, thyroid function tests, and annual chest CT scans. When suspicious nodules are detected on ultrasound, patients are advised to undergo FNA or close monitoring. CEUS is employed to evaluate suspicious lymph nodes and any newly emerging nodules within the ablation zone. The definitive diagnosis is ultimately based on FNA results. For recurrent tumors, multidisciplinary team evaluation determined subsequent treatment (repeat ablation or surgical resection).

### 2.3 Procedures

All ablation procedures were performed by radiologists with over 3 years of specialized experience in thyroid nodule ablation. The detailed procedural steps were consistent with our previous study (15, 16). Here, we primarily focus on describing the technical nuances of the hydrodissection technique.

An 18-G core needle connected to an extension tube and normal saline (NS) was used. Under real-time ultrasound guidance, the puncture needle was advanced layer by layer to the target position, with the tip appropriately placed within the fascial space. NS was then injected. If swelling of the surrounding soft tissues was observed, the needle tip was considered improperly positioned and was adjusted until the fascial space expanded upon NS injection, appearing as an anechoic area on ultrasound. During the ablation procedure, NS was continuously injected to maintain an adequate width of the isolation zone (at least 5 mm). The purpose of hydrodissection was to separate the lesion from adjacent critical structures and organs, thereby reducing the risk of complications. During lymph node ablation, hydrodissection was similarly performed to create a fluid barrier surrounding the target lymph node, with complete circumferential isolation as the optimal endpoint.

### 2.4 Definition

**Figure 2:**
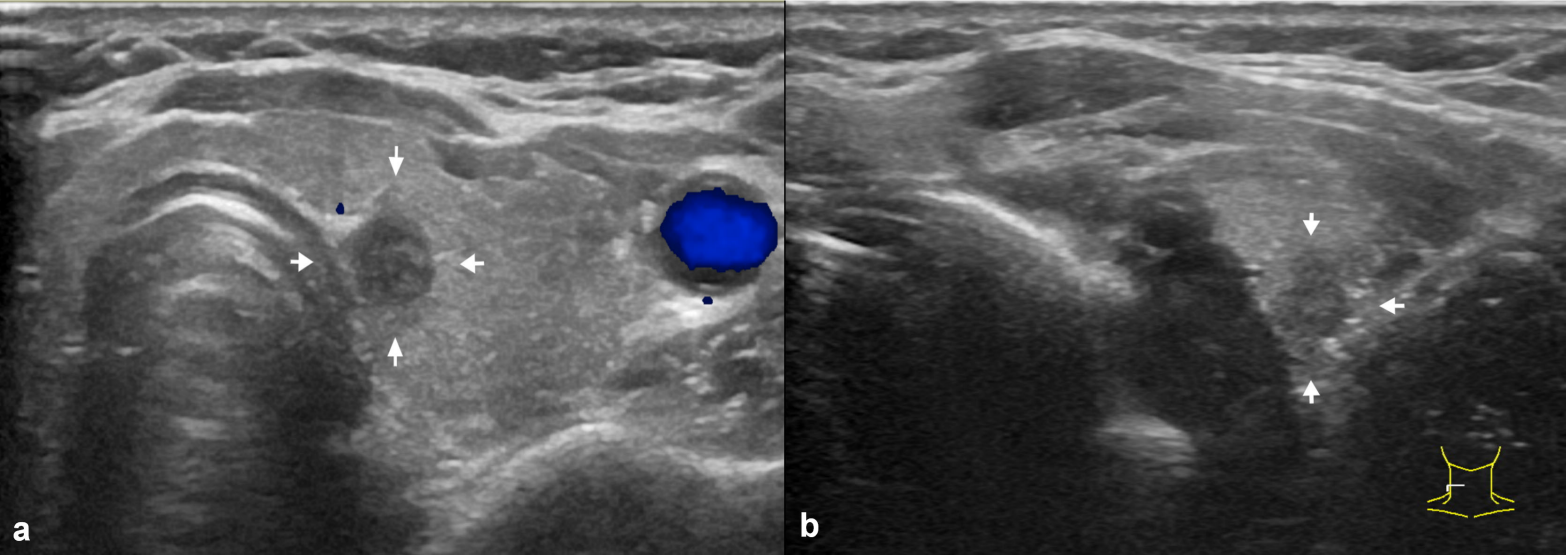
Effective isolation of ultrasound extrathyroidal extension (uETE) nodules. State of the sample before **(a)** and after **(b)** hydrodissection.

Given the nonsurgical nature of thermal ablation (with consequent absence of pathological specimens), and distinct from the surgical-pathological criteria used in the ATA risk stratification system, we established the following sonographic definition of uETE: (1) on ultrasound examination, the tumor shows a tendency to breach the capsule but can be effectively isolated from adjacent soft tissues by hydrodissection. which specifically characterized by the presence of interrupted or defective capsular echoes adjacent to the tumor, (2) but after isolation, a separation of at least 5 mm is maintained between the tumor and surrounding important structures as opposed to mere soft tissue edema (**Figure 2**). Technical success was achieved when the tumor was entirely encompassed by the ablation zone with a minimum 2-mm safety margin (except at the capsular interface). Complete ablation was confirmed by immediate post-procedural CEUS demonstrating no enhancement within the treated area; any residual enhancement prompted additional ablation. RFS was measured from the date of confirmed complete ablation until either disease recurrence or death from any cause. Disease recurrence encompassed: (1) pathologically-confirmed local recurrence (tumor regrowth within or adjacent to the ablation zone), (2) new primary tumors (distinct from the ablation site), (3) emergence of new lymph node metastases, or (4) distant metastasis. Procedure-related complications encompassed both immediate adverse events and subsequent occurrences with probable treatment association, where complications persisting beyond 6 months were designated as permanent (17, 18). Assessment of major complications (hoarseness or permanent·vocal cord paralysis) and minor complications (choking cough, hematoma, shoulder pain). The tumor volume was calculated using the following formula: V=πabc/6, where V represents the volume, A is the largest diameter, B and C are the other two perpendicular diameters.

### 2.5 Statistical analysis

Continuous variables were compared using independent samples t-test or Mann-Whitney U test. Categorical variables were analyzed using Fisher’s exact test, chi-square test or continuity-corrected chi-square test. The Kolmogorov-Smirnov test was used for normality testing. Survival curves were generated by Kaplan-Meier method and compared using log-rank test. A p-value <0.05 was considered statistically significant. Univariate and multivariate regression analyses (Cox regression and logistic regression) were performed to identify prognostic factors, with variables showing p<0.1 in univariate analysis included in multivariate analysis. All analyses were conducted using SPSS (version 25.0, Chicago, USA) and R software (version 4.1.1).

## 3 Results

### 3.1 Characteristics

After applying the inclusion and exclusion criteria, a total of 113 patients were enrolled in this study. Their demographic and tumor characteristics are summarized in **Table 1**. Among them, 94 patients (83.2%) harbored BRAF mutations, while 19 (16.8%) were BRAF wild-type. The median number of ablated nodules was 2 (IQR: 2, 4), and the median number of intrathyroidal tumors was 1 (IQR: 1, 2). The median volume of the index lesion was 0.30 cm³ (IQR: 0.11, 1.05). Regarding tumor characteristics, 77.0% (87/113) of index lesions showed no calcification, while 23.0% (26/113) exhibited calcifications. On CEUS, 48.7% (55/113) demonstrated hyperenhancement, 5.3% (6/113) showed isoenhancement and 46.0% (52/113) presented with hypoenhancement or no enhancement. Lymph node metastasis was observed in 55.8% (63/113) of patients, with a median of 2 metastatic lymph nodes (IQR: 1, 3) and a median maximum diameter of 0.90 cm (IQR: 0.70, 1.45). Additionally, 48.7% (55/113) of patients exhibited uETE, with specific invasion sites detailed in **Table 2**. The median ablation time was 150.0 seconds (IQR: 98.00, 301.00). The mean ablation power was 30–40 W.

**Table 1:** Patient demographics and baseline characteristics.

Characteristic	N = 113^1^
Age	
Median (IQR)	38 (32, 47)
Gender	
Male	74 (65.5%)
Female	39 (34.5%)
Number of ablation lesions	
Median (IQR)	2.00 (2.00, 4.00)
Number of intrathyroidal lesions	
Median (IQR)	1.00 (1.00, 2.00)
Diameter A	
Median (IQR)	1.00 (0.70, 1.50)
Diameter B	
Median (IQR)	0.80 (0.60, 1.10)
Diameter C	
Median (IQR)	0.75 (0.60, 1.10)
Volume	
Median (IQR)	0.30 (0.11, 1.05)
Calcify	
No	87 (77.0%)
Yes	26 (23.0%)
Location	
Right	59 (52.2%)
Left	49 (43.4%)
Isthmus	5 (4.4%)
Lymph node metastasis	
No	50 (44.2%)
Yes	63 (55.8%)
Number of lymph node metastases	
Median (IQR)	2.00 (1.00, 3.00)
Maximum diameter of metastatic lymph nodes	
Median (IQR)	0.90 (0.70, 1.45)
Invasion	
No	58 (51.3%)
Yes	55 (48.7%)
Mutation	
No	19 (16.8%)
Yes	94 (83.2%)
CEUS	
Hyperenhancement	55 (48.7%)
Hypoenhancement	52 (46.0%)
Isoenhancement	6 (5.3%)
Number of ablations	
1	108 (95.6%)
2	5 (4.4%)
Type of ablation	
Microwave	103 (91.2%)
Radiofrequency	10 (8.8%)
Complication	
No	89 (78.8%)
Yes	24 (21.2%)

^1^n (%) IQR, Interquartile Range; CEUS, Contrast-Enhanced Ultrasound.

**Table 2:** Location of PTC invasion.

Location of invasion	N = 55^1^
Medial	15 (27.3%)
Posterior	19 (34.5%)
Anterior	6 (10.9%)
Lateral	3 (5.5%)
Posterior, medial and lateral	1 (1.8%)
Posterior and medial	6 (10.9%)
Anterior and medial	2 (3.6%)
Posterior and lateral	3 (5.5%)

^1^n (%).

### 3.2 Treatment outcomes

A total of 380 nodules, including 194 PTC nodules and 186 metastatic lymph nodes, underwent ablation treatment. Among the 113 patients, 118 ablation sessions were performed, with 108 (95.6%) underwent single ablation session and 5 patients (4.4%) requiring a second session due to either loss of visualization during continuous separating fluid injection in cases with multiple lesions or procedure-related complications. Post-treatment CEUS confirmed the absence of enhancement within all ablation zones, resulting in 100% technical success and complete ablation rates.

### 3.3 Efficacy and prognostic factors

**Figure 3:**
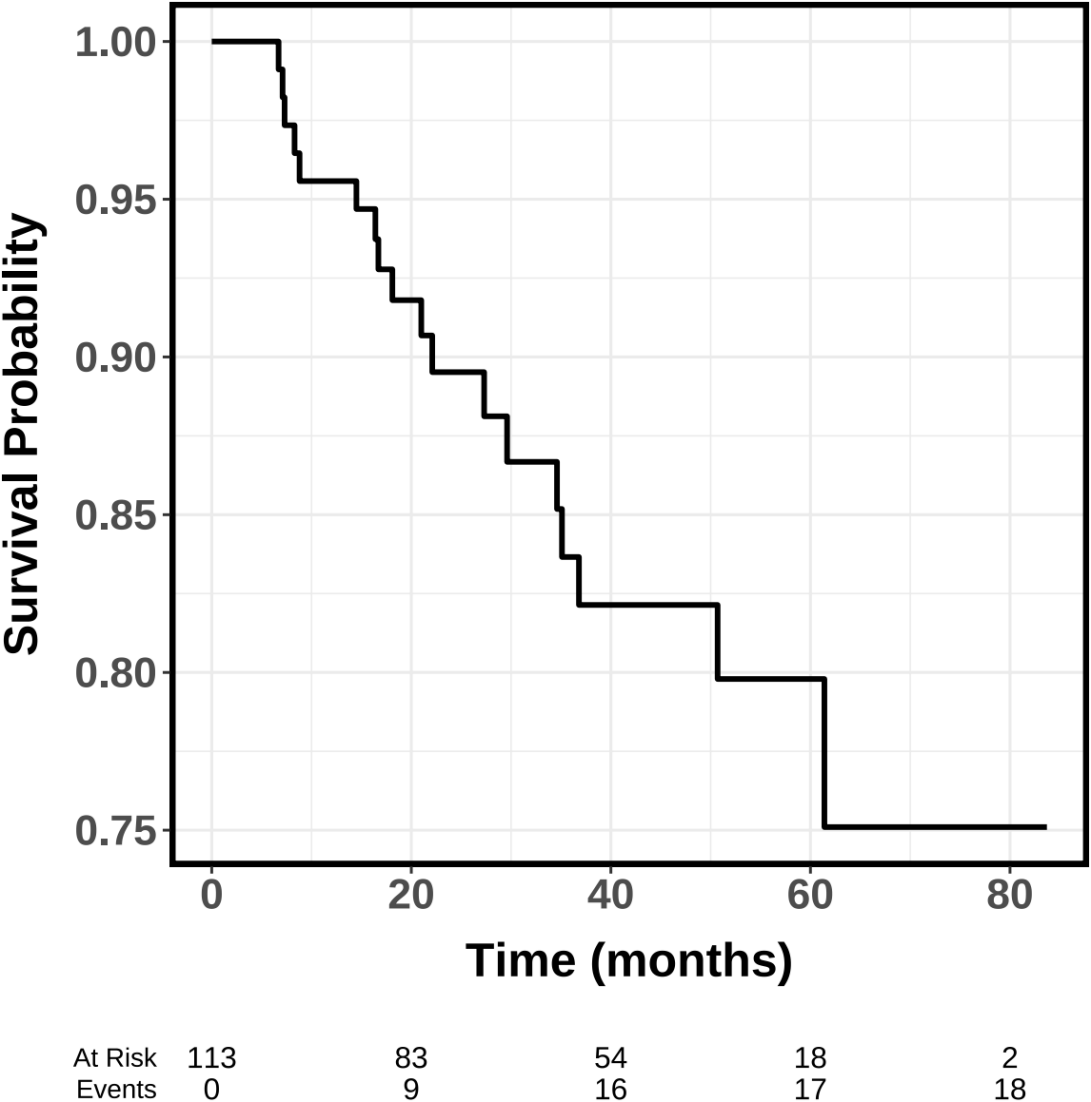
Kaplan-Meier survival curves of TA for ATA intermeidate-risk PTC. TA, thermal ablation; PTC, Papillary thyroid carcinoma.

The median follow-up duration was 45.7 months (95% CI: 34.6–48.7). By the end of follow-up, 18 patients (15.9%) had developed tumor recurrence. Notably, all recurrences manifested as new primary tumors rather than local recurrence. The mean RFS was 70.8 ± 2.8 months, with 1-, 2-, 3-, and 5-year recurrence-free survival rates of 95.6% (95% CI: 91.9%–99.4%), 89.5% (95% CI: 83.8%–95.6%), 83.7% (95% CI: 76.2%–91.9%) and 79.8% (95% CI: 71.1%–89.5%). The survival curve is presented in **Figure 3**. Univariate and multivariate analyses (**Table 3**) identified metastatic lymph node diameter as an independent risk factor for RFS (HR: 3.20, 95% CI: 1.32–7.76, *p* < 0.05).

**Table 3:** Univariate and multivariate analysis of recurrence-free survival.

Characteristic	Univariable					Multivariable				
	N	Event N	HR	95% CI	*P*-value	N	Event N	HR	95% CI	*P*-value
Number of ablations										
1	108	16	—	—						
2	5	2	2.76	0.63, 12.09	0.178					
Gender										
Female	74	13	—	—						
Male	39	5	0.76	0.27, 2.14	0.608					
**Age**	113	18	1.00	0.96, 1.04	0.932					
Mutation										
No	19	2	—	—						
Yes	94	16	1.64	0.38, 7.13	0.511					
**Type of ablation**										
Microwave	103	14	—	—		103	14	—	—	
Radiofrequency	10	4	3.20	1.04, 9.84	**0.042**	10	4	1.91	0.53, 6.91	0.325
**Number of ablation lesions**	113	18	1.08	0.97, 1.21	0.137					
**Number of intrathyroidal lesions**	113	18	0.76	0.44, 1.31	0.328					
Location										
Isthmus	5	1	—	—						
Left	49	11	1.20	0.15, 9.33	0.862					
Right	59	6	0.58	0.07, 4.84	0.613					
**Volume**	113	18	1.11	0.87, 1.42	0.400					
Lymph node metastasis										
No	50	2	—	—		50	2	—	—	
Yes	63	16	3.98	0.89, 17.80	0.070	63	16	0.88	0.12, 6.36	0.903
Calcify										
No	87	15	—	—						
Yes	26	3	0.89	0.25, 3.10	0.850					
CEUS										
Hyperenhancement	55	7	—	—						
Isoenhancement	6	1	1.13	0.14, 9.18	0.910					
Hypoenhancement	52	10	1.26	0.48, 3.34	0.641					
Invasion										
No	58	14	—	—						
Yes	55	4	0.48	0.15, 1.50	0.205					
**Number of lymph node metastases**	113	18	1.11	1.00, 1.24	0.049	113	18	1.00	0.85, 1.18	0.996
**Maximum diameter of MLN**	113	18	3.36	1.74, 6.49	**<0.001**	113	18	3.20	1.32, 7.76	**0.010**
Complication										
No	89	14	—	—						
Yes	24	4	0.94	0.31, 2.84	0.906					

HR, Hazard Ratio; CI, Confidence Interval; CEUS, Contrast-Enhanced Ultrasound; MLN, metastatic lymph nodes. The bold values indicate statistical significance (p < 0.05).

### 3.4 Complications and associated risk factors

By the end of follow-up, 24 patients (21.2%) had experienced procedure-related complications (**Table 4**). Major complications occurred in 21 (18.6%) patients, while minor complications in 8 (7.1%). To elaborate in detail, the most frequent complication was hoarseness (18.6%, 21/113), including 2 cases (1.8%) of permanent vocal cord paralysis. Other complications included choking cough [occurs concomitantly with hoarsenes (4.4%, 5/113)], hematoma formation (1.8%, 2/113) and shoulder pain (0.9%, 1/113). All complications except permanent hoarseness resolved completely within 3 months post-procedure. Univariate and multivariate analyses (**Table 5**) revealed that number of ablation sessions was independently associated with complication occurrence (OR: 13.54, 95% CI: 1.14–160.19, *p* < 0.05).

**Table 4:** Complication.

Complication	N
Major complications	
Transient hoarseness	19 (16.8%)
Permanent hoarseness	2 (1.8%)
Minor complication	
Choking cough	5 (4.4%)
Hematoma	2 (1.8%)
Shoulder pain	1 (0.9%)

**Table 5:** Univariate and multivariate analysis of complication.

Characteristic	Univariable					Multivariable				
	N	Event N	OR	95% CI	P-value	N	Event N	OR	95% CI	P-value
Number of ablation										
1	108	20	—	—		108	20	—	—	
2	5	4	17.60	1.87, 166.06	**0.012**	5	4	13.54	1.14, 160.19	**0.039**
Gender										
Female	74	17	—	—						
Male	39	7	0.73	0.28, 1.96	0.536					
**Age**	113	24	1.02	0.98, 1.06	0.374					
Mutation										
No	19	5	—	—						
Yes	94	19	0.71	0.23, 2.21	0.554					
Type of ablation										
Microwave	103	19	—	—		103	19	—	—	
Radiofrequency	10	5	4.42	1.16, 16.81	**0.029**	10	5	3.74	0.83, 16.92	0.086
**Number of ablation lesions**	113	24	1.18	1.02, 1.36	**0.023**	113	24	1.01	0.61, 1.67	0.983
**Number of intrathyroidal lesions**	113	24	1.11	0.70, 1.76	0.662					
Location										
Isthmus	5	2	—	—						
Left	49	13	0.54	0.08, 3.62	0.527					
Right	59	9	0.27	0.04, 1.85	0.182					
**Volume**	113	24	1.28	0.93, 1.76	0.133					
Calcify										
No	87	20	—	—						
Yes	26	4	0.61	0.19, 1.98	0.409					
Lymph node metastasis										
No	50	9	—	—						
Yes	63	15	1.42	0.56, 3.59	0.455					
CEUS										
Hyperenhancement	55	10	—	—						
Isoenhancement	6	1	0.90	0.09, 8.57	0.927					
Hypoenhancement	52	13	1.50	0.59, 3.80	0.392					
Invasion										
No	58	13	—	—						
Yes	55	11	0.87	0.35, 2.14	0.754					
**Number of lymph node metastases**	113	24	1.20	1.03, 1.40	**0.021**	113	24	1.05	0.60, 1.82	0.873
**Maximum diameter of MLN**	113	24	1.46	0.78, 2.75	0.239					

HR, Hazard Ratio; CI, Confidence Interval; CEUS, Contrast-Enhanced Ultrasound; MLN, metastatic lymph nodes. The bold values indicate statistical significance (p < 0.05).

## 4 Discussion

This pioneering study provides the first comprehensive assessment of TA for ATA “intermediate-risk” PTC, demonstrating that TA is a technically feasible approach for this population, with excellent procedural success rates (both technical success and complete ablation rates of 100%). Additionally, TA provided acceptable long-term oncological outcomes, with a 5-year recurrence-free survival rate of 79.8%, and a manageable complication profile (21.2% overall complication rate). Our analysis also identified metastatic lymph node diameter as a significant prognostic factor for disease progression. This finding is consistent with previous studies reporting that TA is a safe approach for managing lymph node metastases, although the size of the metastatic lymph nodes may influence treatment outcomes (19). Furthermore, the size of metastatic lymph nodes is also recognized in the ATA guidelines as an important prognostic factor for surgical outcomes (13, 14).

Previous research has predominantly focused on TA applications in low-risk thyroid carcinoma such as solitary microcarcinomas (8, 12, 20, 21), while evidence for intermediate-risk cases remains limited. Although surgical resection remains the cornerstone of curative treatment for thyroid cancer, it still carries a 10%-30% recurrence risk for ATA intermediate-risk patients (13, 14), along with significant treatment-related burdens such as lifelong hormone replacement therapy, permanent surgical scarring and increased complication risks from reoperations due to fibrotic changes (16, 22, 23). Our findings preliminarily demonstrate that TA achieves comparable long-term recurrence-free survival to conventional surgery in selected ATA intermediate-risk patients. Importantly, accumulating evidence suggests TA offers reduced tissue trauma, lower treatment costs and preservation of thyroid function over surgery (24–26). These results collectively suggest TA may represent a viable minimally invasive alternative for ATA intermediate-risk PTC patients.

An important and potentially contentious aspect of our study lies in the definition of uETE. While patients with capsular echo disruption may not strictly meet pathological criteria for mETE (13, 14, 27), the feasibility of successful separation in imaging is both visually intuitive and highly significant. Capsular echo defects or even interruptions indicate the potential for the originally intact capsular structure to be invaded by tumor cells. Our previous studies have demonstrated that the perithyroidal region contains abundant and complex fascial spaces (28). However, the ability to achieve effective separation by isolating fluid suggests a less severe degree of infiltration, implying that normal fascial planes between the thyroid and adjacent structures can be preserved without significant adhesion formation. Given the expanding application of TA technology in thyroid nodules and the in-depth imaging study in the field of anatomy, establishing an imaging-based stratification system is crucial for patients who do not opt for surgical treatment. However, based on the current situation, the ATA stratification system was specifically designed for surgical candidates, incorporating many pathological parameters obtainable only after resection. In the absence of ablation-specific criteria, we employed customized ultrasonographic standards to evaluate TA’s therapeutic effects in this population provisionally. We acknowledge this represents a cautious but necessary step forward, and we are actively working to establish dedicated risk stratification criteria for the non-surgical patient cohorts.

Regarding complication rates, studies have shown that thyroid surgery-specific complications (including only permanent hypoparathyroidism/hypocalcemia and vocal cord/fold paralysis) occur in 15.7% of patients with locoregional disease, with lymph node dissection associated with higher complication rates (29) An earlier study reported that thyroidectomy resulted in vocal cord paralysis in 13.7% of patients with regional extension and 22.4% of those with both regional extension and lymph node metastasis in well-DTC and among all patients experiencing vocal cord paralysis, 22% required additional surgical intervention specifically for this complication (30). In this study, permanent hoarseness occurred in 2 patients (1.8%), while all other complications resolved within 3 months post-procedure. Prior studies have demonstrated that nodule volume, location, RFA and ablation power are independent factors associated with the incidence of complications in thyroid nodule ablation (31, 32). In the present study, given that all enrolled patients had nodules adjacent to the thyroid capsule, the sample size was limited. Univariate analysis revealed an association between RFA and the occurrence of complications, that may be attributed to the inherent characteristics of RFA. Multivariate analysis further identified the number of ablation sessions as an independent factor correlated with the complication rate. This correlation may be attributed to alterations in the thyroid’s anatomical microenvironment following ablation. Multiple ablation procedures could lead to localized structural changes and even adhesion formation within the thyroid, a phenomenon that has been documented in previous studies (33).

This study has several limitations that should be acknowledged: (1) as a retrospective analysis, the inherent selection bias may affect the reliability of our findings; (2) the relatively small sample size prevented meaningful subgroup analyses, and larger prospective studies are needed to establish more detailed ablation protocols.

## 5 Conclusion

Thermal ablation for ATA intermediate-risk PTC patients demonstrates high technical feasibility, with 1-, 2-, 3-, and 5-year recurrence-free survival rates of 95.6%, 89.5%, 83.7%, and 79.8% respectively. The overall complication rate was 21.2%, and metastatic lymph node diameter was identified as an independent risk factor for PFS. These results confirm that TA is an effective and safe treatment for ATA intermediate-risk PTC patients.

## Data Availability

The raw data supporting the conclusions of this article will be made available by the authors subject to reasonable request, without undue reservation.

## References

[1] Baloch ZW Asa SL Barletta JA Ghossein RA Juhlin CC Jung CK . Overview of the 2022 WHO classification of thyroid neoplasms. Endocr Pathol. (2022) 33:27–63. doi: 10.1007/s12022-022-09707-3, PMID: 35288841

[2] Lohia S Hanson M Tuttle RM Morris LGT . Active surveillance for patients with very low-risk thyroid cancer. Laryngoscope Investig Otolaryngol. (2020) 5:175–82. doi: 10.1002/lio2.356, PMID: 32128446 PMC7042648

[3] Aschebrook-Kilfoy B Ward MH Sabra MM Devesa SS . Thyroid cancer incidence patterns in the United States by histologic type, 1992-2006. Thyroid. (2011) 21:125–34. doi: 10.1089/thy.2010.0021, PMID: 21186939 PMC3025182

[4] Arora N Turbendian HK Scognamiglio T Wagner PL Goldsmith SJ Zarnegar R . Extrathyroidal extension is not all equal: Implications of macroscopic versus microscopic extent in papillary thyroid carcinoma. Surgery. (2008) 144:942–8. doi: 10.1016/j.surg.2008.07.023, PMID: 19041001

[5] Chung SR Baek JH Choi YJ Sung TY Song DE Kim TY . Risk factors for metastasis in indeterminate lymph nodes in preoperative patients with thyroid cancer. Eur Radiol. (2022) 32:3863–8. doi: 10.1007/s00330-021-08478-5, PMID: 34989848

[6] Davies L Hoang JK . Thyroid cancer in the USA: current trends and outstanding questions. Lancet Diabetes Endocrinol. (2021) 9:11–2. doi: 10.1016/S2213-8587(20)30372-7, PMID: 33220765

[7] Tufano RP Pace-Asciak P Russell JO Suarez C Randolph GW Lopez F . Update of radiofrequency ablation for treating benign and Malignant thyroid nodules. The future is now. Front Endocrinol (Lausanne). (2021) 12:698689. doi: 10.3389/fendo.2021.698689, PMID: 34248853 PMC8264548

[8] Kim JH Baek JH Lim HK Ahn HS Baek SM Choi YJ . 2017 Thyroid radiofrequency ablation guideline: korean society of thyroid radiology. Korean J Radiol. (2018) 19:632–55. doi: 10.3348/kjr.2018.19.4.632, PMID: 29962870 PMC6005940

[9] Xu D Ge M Yang A Cheng R Sun H Wang H . Expert consensus workshop report: Guidelines for thermal ablation of thyroid tumors (2019 edition). J Cancer Res Ther. (2020) 16:960–6. doi: 10.4103/jcrt.JCRT_558_19, PMID: 33004735

[10] Mauri G Hegedus L Bandula S Cazzato RL Czarniecka A Dudeck O . European thyroid association and cardiovascular and interventional radiological society of europe 2021 clinical practice guideline for the use of minimally invasive treatments in Malignant thyroid lesions. Eur Thyroid J. (2021) 10:185–97. doi: 10.1159/000516469, PMID: 34178704 PMC8215982

[11] Durante C Hegedus L Czarniecka A Paschke R Russ G Schmitt F . 2023 European Thyroid Association Clinical Practice Guidelines for thyroid nodule management. Eur Thyroid J. (2023) 12. doi: 10.1530/ETJ-23-0067, PMID: 37358008 PMC10448590

[12] Hu Y Zhou W Xu S Jia W Zhang G Cao Y . Thermal ablation for the treatment of Malignant thyroid nodules: present and future. Int J Hyperthermia. (2024) 41:2379983. doi: 10.1080/02656736.2024.2379983, PMID: 39013550

[13] Haugen BR . 2015 American Thyroid Association Management Guidelines for Adult Patients with Thyroid Nodules and Differentiated Thyroid Cancer: What is new and what has changed? Cancer. (2017) 123:372–81. doi: 10.1002/cncr.30360, PMID: 27741354

[14] Ringel MD Sosa JA Baloch Z Bischoff L Bloom G Brent GA . 2025 American thyroid association management guidelines for adult patients with differentiated thyroid cancer. Thyroid®. (2025) 35:841–985. doi: 10.1177/10507256251363120, PMID: 40844370 PMC13090833

[15] Zhou HD Yu XY Wei Y Zhao ZL Peng L Li Y . A preliminary study on the microwave ablation of multifocal papillary thyroid microcarcinoma. Acad Radiol. (2024) 31:2306–11. doi: 10.1016/j.acra.2024.01.007, PMID: 38262812

[16] Zhao ZL Wang SR Dong G Liu Y He JF Shi LL . Microwave ablation versus surgical resection for US-detected multifocal T1N0M0 papillary thyroid carcinoma: A 10-center study. Radiology. (2024) 311:e230459. doi: 10.1148/radiol.230459, PMID: 38563669

[17] Patel KN Yip L Lubitz CC Grubbs EG Miller BS Shen W . The american association of endocrine surgeons guidelines for the definitive surgical management of thyroid disease in adults. Ann Surg. (2020) 271:e21–93. doi: 10.1097/SLA.0000000000003580, PMID: 32079830

[18] Lombardi CP Carnassale G Damiani G Acampora A Raffaelli M De Crea C . The final countdown”: Is intraoperative, intermittent neuromonitoring really useful in preventing permanent nerve palsy? Evidence from a meta-analysis. Surgery. (2016) 160:1693–706. doi: 10.1016/j.surg.2016.06.049, PMID: 27566947

[19] Xiao X Chen X Li J Li P Zhu Y . Microwave ablation for lymph node metastasis in thyroid cancer: the impact of lymph node diameter. Front Endocrinol (Lausanne). (2024) 15:1430693. doi: 10.3389/fendo.2024.1430693, PMID: 39165510 PMC11333885

[20] Ledesma-Leon T Solis-Pazmino P Lincango EP Figueroa LA Ellenhorn J Nasseri Y . Ablation techniques or active surveillance compared to surgical resection in patients with low-risk papillary thyroid cancer: a systematic review and meta-analysis. Endocrine. (2024) 83:330–41. doi: 10.1007/s12020-023-03502-8, PMID: 37658978

[21] He H Song Q Lan Y Yan L Xiao J Zhang Y . Efficacy and safety of ultrasound-guided radiofrequency ablation for low-risk papillary thyroid microcarcinoma in patients aged 55 years or older: a retrospective study. Int J Hyperthermia. (2021) 38:604–10. doi: 10.1080/02656736.2021.1912416, PMID: 33853489

[22] Burman KD . Treatment of recurrent or persistent cervical node metastases in differentiated thyroid cancer: deceptively simple options. J Clin Endocrinol Metab. (2012) 97:2623–5. doi: 10.1210/jc.2012-2480, PMID: 22869846

[23] Tufano RP Mohamed Ali K . The year in surgical thyroidology: recent technological developments and future challenges. Thyroid. (2022) 32:14–8. doi: 10.1089/thy.2021.0590, PMID: 34915767

[24] Wei Y Niu WQ Zhao ZL Wu J Peng LL Li Y . Microwave ablation versus surgical resection for solitary T1N0M0 papillary thyroid carcinoma. Radiology. (2022) 304:704–13. doi: 10.1148/radiol.212313, PMID: 35536133

[25] Cao XJ Wang SR Che Y Liu J Cong ZB He JF . Efficacy and safety of thermal ablation for treatment of solitary T1N0M0 papillary thyroid carcinoma: A multicenter retrospective study. Radiology. (2021) 300:209–16. doi: 10.1148/radiol.2021202735, PMID: 33904775

[26] Shen K Xue S Xie Y Wang H Li J Sun Y . Comparison of thermal ablation and routine surgery for the treatment of papillary thyroid microcarcinoma: a systematic review and Meta-analysis. Int J Hyperthermia. (2020) 37:913–24. doi: 10.1080/02656736.2020.1777331, PMID: 32722973

[27] Bortz MD Kuchta K Winchester DJ Prinz RA Moo-Young TA . Extrathyroidal extension predicts negative clinical outcomes in papillary thyroid cancer. Surgery. (2021) 169:2–6. doi: 10.1016/j.surg.2020.04.003, PMID: 32682508

[28] Wei Y Zhao Z-l Niu Y Peng L-l Li Y Yu M-A . Ultrasound imaging of the perithyroid fascial space: a comparative analysis with anatomical correlations. Sci Rep. (2025) 15:4503. doi: 10.1038/s41598-025-88306-8, PMID: 39915549 PMC11803094

[29] Papaleontiou M Hughes DT Guo C Banerjee M Haymart MR . Population-based assessment of complications following surgery for thyroid cancer. J Clin Endocrinol Metab. (2017) 102:2543–51. doi: 10.1210/jc.2017-00255, PMID: 28460061 PMC5505192

[30] Francis DO Pearce EC Ni S Garrett CG Penson DF . Epidemiology of vocal fold paralyses after total thyroidectomy for well-differentiated thyroid cancer in a Medicare population. Otolaryngol Head Neck Surg. (2014) 150:548–57. doi: 10.1177/0194599814521381, PMID: 24482349 PMC4229384

[31] Liang X Jiang B Ji Y Xu Y Lv Y Qin S . Complications of ultrasound-guided thermal ablation of thyroid nodules and associated risk factors: an experience from 9667 cases. Eur Radiol. (2025) 35:2307–19. doi: 10.1007/s00330-024-11023-9, PMID: 39174654

[32] Wu S Cai Y Liu X Zhu C . Complications after thermal ablation for thyroid nodules across countries. Front Endocrinol (Lausanne). (2025) 16:1608164. doi: 10.3389/fendo.2025.1608164, PMID: 40801030 PMC12339322

[33] Chung SR Baek JH Choi YJ Lee JH . Longer-term outcomes of radiofrequency ablation for locally recurrent papillary thyroid cancer. Eur Radiol. (2019) 29:4897–903. doi: 10.1007/s00330-019-06063-5, PMID: 30805701

